# Evaluation of Fast Food Behavior in Pre-School Children and Parents Following a One-Year Intervention with Nutrition Education 

**DOI:** 10.3390/ijerph110706780

**Published:** 2014-06-30

**Authors:** Yongqing Gao, Yuee Huang, Yongjun Zhang, Fengqiong Liu, Cindy Xin Feng, Tingting Liu, Changwei Li, DongDong Lin, Yongping Mu, Siobhan L. Tarver, Mao Wang, Wenjie Sun

**Affiliations:** 1 School of Food Science, Guangdong Pharmaceutical University, Zhongshan 528458, China; E-Mail: yongqingg@163.com; 2 School of Public Health, Wannan Medical University, Wuhu 241001, China; E-Mail: huangyewindow@163.com; 3 Yijishan Hospital of Wannan Medical University, Wuhu 241001, China; E-Mail: Zyj200888@tom.com; 4 School of Public Health, Sun Yatsen University, Guangzhou 510080, China; E-Mail: meimei20061986@126.com; 5 School of Public Health, University of Saskatchewan, Saskatoon SK S7N 5E5, Canada; E-Mail: cindy.feng@usask.ca; 6 Nell Hodgson Woodruff School of Nursing, Emory University, Atlanta, GA 30322, USA; E-Mail: tliu26@emory.edu; 7 School of Public Health and Tropical Medicine, Tulane University, New Orleans, LA 70112, USA; E-Mails: cli8@tulane.edu (C.L.); starver1@tulane.edu (S.T.); 8 School of Science and Engineering, Tulane University, New Orleans, LA 70112, USA; E-Mail: dlin5@tulane.edu; 9 The Affiliated People’s Hospital of Inner Mongolia Medical University, Hohhot 010110, China; E-Mail: ypmu040@sina.com

**Keywords:** western style fast food, Chinese parents, young children

## Abstract

A community-based intervention study was conducted to assess a nutrition education intervention on western style fast food consumption among Chinese children and parents. Eight kindergartens from three district areas of Hefei City (a total of 1252 children aged 4–6 years and their parents) were randomly selected. Descriptive and analytical statistical methods were used to evaluate the baseline, midterm, and final western style fast food knowledge, attitude, and practice in both parents and children were used to identify and compare the knowledge, attitude, and practice in the parents and children. Parents and children were divided into “intervention” and “control” groups based on nutrition education status. Consumption of western style fast food at breakfast in Chinese children and parents is not high. The main reasons for this in children is that consumption of western style fast food is not viewed as “food”, but rather as a “gift” or “interesting”. The time of children’s consumption of western style fast food is mostly likely to be in the weekends. The nutrition education modified the parents’ western style fast food behavior (*p* < 0.01), although it did not change significantly in children. The healthy nutrition concept should be built up among Chinese, especially in children. Insights from the families provide leads for future research and ideas for the nutrition education.

## 1. Introduction

There has been a dramatic rise of overweight and obesity among young Chinese children, according to the data from three cross-sectional National Nutrition and Health Surveys [1]. The rapidly increasing trend in obesity was attributed to unhealthy diet behaviors, *i.e.*, consumption of greater quantities of energy-dense, low-nutrient foods [2,3,4]. “Obesogenic environment is an environment that promotes gaining weight and one that is not conducive to weight loss” within the home or workplace [5]. The obesogenic enivorment in China is building. For example, KFC boasted 4,260 locations in only 26 years in China while it took 61 years to amass 4,618 location in the USA [6]. An English study showed that children living near fast food outlets are more likely to be overweight [7].

Fast-food consumers were more likely to exceed the Recommended Dietary Allowances for energy, fat and saturated fat, and less likely to meet wholegrain and fruit recommendations [8]. That might be due to the fact that people eating at fast food restaurants tend to underestimate the calorie content of meals, especially large meals [9]]. western style fast food intake was associated with increased risk of developing type 2 diabetes mellitus and of coronary heart disease mortality in an Eastern population [10]. According to study based on China Health and Nutrition Survey, Xu *et al.*, pointed out that the associations between community exposure to western style fast food and weight changes are temporally dynamic rather than static [11]. Zhang *et al*. claimed the irrelevance of the connection between fast-food consumption and overweight/obesity for Chinese segments that do not have access to fast food. Factors that are most associated with obesity in segments with a higher Body Mass Index are consumers’ (incorrect) dietary knowledge, the food retail environment and socio-demographics. They also suggested that despite the breathtaking changes in modern China, the impact of “obesogenic” environments should not be assessed too strictly from a “Western” perspective [12]. It is a pressing public health concern and highlights for the need of early prevention. However, in their study, participants are adults aged 18 years or above whose “diet pattern” and attitudes have already established and are harder to change. 

Children, especially young children, are more vulnerable to the environment and easily attracted by an unhealthy diet pattern [13]. Once they have established these unhealthy dietary patterns during their early years, it is hard to correct in later life. Unfortunately, unlike people in western countries, Chinese did not realize the potential risks of western style fast food. Hence, modifying the obesogenic environment at the community level is urgent. These changes could include access to healthy food and access to places to be physically active; thereby, supporting an individual’s healthy behaviors.

Nutrition education has been linked to diet behavior while the relative western style fast food behavior in Chinese children and parents remains unclear. Hence, we conducted the present study to find the western style fast food consumption patterns in Chinese children and parents and determine whether nutrition education would change the parents’ and children’s western style fast food behavior. 

The aim of the present study is thus to investigate western style fast food consumption in young Chinese children and their parents and whether fast-food consumption can be modified by nutrition education. We hypothesized that fast-food consumption can be modified in both Chinese children and parents by appropriate nutrition education. 

## 2. Methods

Briefly, the present study reports the results of a 2-year intervention study started in September 2001, which recruited children aged 4–6 years old and their respective parent pairs from eight kindergartens in Hefei City (the capital of Anhui Province, China) of 2012. Hefei City was divided into three administrative districts: east urban, central urban and west urban, which contain a total of seventeen kindergartens (8,752 children in total) to which parents send the children in the morning. Eight kindergartens were selected by stratified cluster sampling from all the kindergartens in each three districts, according to the population density in each area. Kindergartens with a small sample size (less than 200 children) were excluded. Randomization was stratified by administrative district in order to achieve heterogeneity in each location; the kindergartens were then randomized to either an intervention group (five kindergartens and 1,252 child-parent pairs) or a control group (three kindergartens and 850 child-parent pairs) using computer-generated numbers within each district. Informed consent forms were obtained from 92.3% of the families who agreed to participate in the project after the purpose of the study was explained.

## 3. Data Collection

At baseline, social and demographic information (e.g., age, gender, parental education, family income) were collected. A questionnaire on western style fast food habits, which demonstrated to be both feasible and reliable, was used to record western style fast food behaviors. The parents recorded both the children and parent’s western style fast food behavior (frequency, time, and reason). Instructions were provided to parents on how to properly record the consumption of any foods or drinks. The questionnaires were reviewed by the study authors for completeness and accuracy of data collection. All questionnaires were completed by the children’s parents.

### 3.1. Baseline Surveys

Social and demographic information and data of western style fast food behaviors of children and their parents was collected in the intervention group and the control group. Table 1 show the details of the sampling. 

### 3.2. Implementation of Early Nutrition Education

In the intervention day-care centers, nutrition instructions were given to parents, who were trained with nutritional knowledge periodically and throwaway were delivered to them; a nutrition wall map was pasted and children received nutrition education, which lasted for two semesters.

According to established teaching procedures, nutrition education is incorporated into the academically oriented curriculum. Information about foods and menus as learning tools used for basic nutritional education were incorporated into teaching activities including language, science, arts, math, music and physical education (motor development).

**Table 1:** Components in intervention and control groups

Group	Site	Number of Subjects	Location
Intervention	Kindergarten affiliated to the organ of 4th engineering bureau of the Railway Ministry	266	West urban Hefei district
	Kindergarten affiliated to telegraphic bureau of Hefei	180	West urban Hefei district
	Kindergarten affiliated to Anhui Medical University	106	West urban Hefei district
	Suzhou road kindergarten	430	Center urban Hefei district
	The 1st kindergarten affiliated to Hefei Steel company	270	East urban Hefei district
**Subtotal**		1252	
Control	Kindergarten affiliated to Hefei Technical University	280	West urban Hefei district
	Kindergarten affiliated to the organ of the Hefei city Government	390	Center urban Hefei district
	The 2nd kindergarten affiliated to Hefei Steel Company	180	East urban Hefei district
**Subtotal**		850	
**Total**		2102	

A flexible curriculum for in-kindergarten education was delivered monthly to children and parents by nutritionists. The curriculum was developed by nutrition professionals and included basic nutritional information, based on the National Dietary Guidelines for China. Parents were informed of the events by their children’s teachers and training took place within the kindergartens. 

An illustrated book was distributed by teachers to all of the children. The intervention group received a book with a nutritional and western style fast food behavior theme while the control group received a book of general picture stories. As teachers told the stories related to the content of the book, the intervention group children received significant amounts of information regarding nutrition and healthy western style fast food behaviors.

Pamphlets giving nutritional information and describing healthy eating behaviors were delivered to each parent pair included in the intervention group at the beginning of the intervention since the parents are responsible for the daily diet of the child. Parents were instructed to read the pamphlet and were periodically interviewed by the authors. The nutritional prejudices of the parents were addressed directly in a series of activities.

Two series of promotional pictures providing information concerning nutrition, the most common unhealthy western style fast food behaviors were displayed in the intervention group kindergartens throughout the intervention, one series per semester.

The last date of follow-up or censor date was June, 2002. We analyzed the results after two different follow up durations: (a) December 2001 and (b) June 2002. We have presented results from mild term and final stage follow-ups.

### 3.3. Evaluation of Outcomes

SAS for Windows Statistical Software Package Version 8.2 (SAS Institute, Evaluation of a Nutrition Education Intervention 255, Cary, NC, USA) was used for data processing and analysis. A self-control study (longitudinal) is performed within groups and a comparative study (cross-sectional) is performed between groups. Enumeration data are analyzed by χ^2^ test and measurement data are analyzed by the methods of Student *t* test and analysis of variance. western style fast food behaviors of both children and parents at baseline, in the intermediate stage and at the final stage groups were tested. The final intervention group and the control group are used to evaluate the outcomes. The difference between intervention and control groups in the different stages also was addressed. Linear trend test and Kruskal-Waillis test were used for the self-comparison among different time points in the intervention group. All the tests were two sided and significance level was set at 0.05.

## 4. Results

### 4.1. Demographic and Socio-Economic Characteristics

There were no significant differences between intervention group and control group children in gender and family incomes. The age of control group children is slightly higher than that of the intervention group. The demographic and socio-economic characteristics of all subjects are presented in Table 2.

**Table 2:** Characteristics of the intervention and control groups in baseline (mean ± SD).

	Intervention	Control	*p* value
Sex ratio (Boy/Girl)	651/601	456/394	0.55
Age (Years)	4.8 ± 0.7	5.2 ± 1.0	<0.01
Family income/month (RMB *)	1914.2 ± 1256.2	1865.1 ± 1504.5	0.53

Note: * (1 $ = 6.75 RMB).

### 4.2. Effect of Nutrition Education on Dietary Behavior of Children

Among all the children included, the constituents of western style fast food consumption frequency was similar between the intervention group and control group, with the majority of children consuming western style fast food occasionally or seldom. Only 6.4% children in the intervention group and 6.0% in control group consumed western style fast food frequently, as seen in Table 3. 

**Table 3:** Consumption of western-style food among children in Hefei (%).

Group	Phase	Often	Sometimes	Occasionally	Seldom
Intervention	Initial	6.4	19.0	46.8	27.8
	Metaphase	3.8	20.5	52.1	23.5
	Final	4.3	22.4	47.6	25.7
Control	Initial	6.0	18.4	44.9	30.7
	Metaphase	3.1	14.1	45.5	37.2
	Final	1.0	19.6	50.0	29.4

Notes: Often refer >1/week; Sometimes refer <1/week but >1/month; Occasionally <1/month, but >1/year; Seldom < 1/year.

Among the children who consumed western style fast food, 94.7% in the intervention group and 96.5% in the control group consumed western style fast food 1–4 times per month, as listed in Table 4. 

**Table 4:** Frequency of western-style food monthly among children in Hefei (%).

Group	Phase	1	2	3	4	5	6	7	8	9 or More
Intervention	Initial	41.6	31.8	11.3	10.0	2.8	1.3	0.3	1.0	0.3
	Metaphase	42.3	33.9	11.7	7.7	2.0	0.8	0.0	0.4	1.2
	Final	46.0	34.0	11.0	5.5	1.8	0.6	0.0	0.6	0.3
Control	Initial	39.2	34.2	14.6	8.5	2.5	0.0	0.0	0.0	1.0
	Metaphase	47.4	30.3	6.6	13.2	2.6	0.0	0.0	0.0	0.0
	Final	50.0	25.6	17.4	5.8	1.2	0.0	0.0	0.0	0.0

Nutrition education didn’t significantly affect the children’s behavior in consuming western style fast food, as both the percentage of children who frequently consumed western style fast food and the frequency of children consuming western style fast food were similar at the base line, mid-term and final stages of the intervention in the intervention group.

According to our survey, the main motivations driving children to consume western style fast food were for the gifts that were offered with meals, interesting dining environment and for the taste of food. Being requested is the most frequent occasions when parents allowed children to consume western style fast food, followed by weekends, encouragement and birthdays. Nutrition education didn’t change the motivations and occasion of consuming western style fast food according to the results shown in Table 5 and Table 6.

**Table 5:** Reasons of children consuming western-style fast foods (%)

Group	Phase	Taste	For a gift offered	Peer pressure	Interesting	Other
Intervention	Initial	24.6	29.9	6.7	31.5	7.1
	Metaphase	23.8	33.6	6.4	28.4	7.8
	Final	23.1	34.6	6.4	28.4	7.5
Control	Initial	20.9	30.9	6.0	35.7	6.6
	Metaphase	22.3	31.1	8.1	29.1	9.5
	Final	18.9	33.5	9.1	32.9	5.5

**Table 6:** The time for consuming western-style foods (%)

Group	Phase	Birthday	Weekend	Request	Encouragement	Other
Intervention	Initial	9.7	31.7	32.7	16.8	9.2
	Metaphase	9.5	29.0	33.2	17.3	11.0
	Final	12.1	25.9	31.7	18.9	11.4
Control	Initial	11.3	31.9	32.8	15.9	8.1
	Metaphase	14.5	27.6	35.5	13.2	9.2
	Final	13.1	24.4	33.3	20.8	8.3

### 4.3. Effects of Nutrition Education on Dietary Practices of Parents

The results of parents’ western style fast food behavior at baseline, mid-term and post-test are shown in Table 7 and Table 8. Among those who consumed western style fast food, most of them (95.2% in the intervention group and 96.3% in the control group at baseline) consumed western style fast food 1–4 times per month. Nutrition education didn’t significantly change the frequency of western style fast food consumption among people who consumed western style fast food, as shown in Table 8. However, the percentage of parents who frequently consumed western-style foods (Table 7) decreased from 13.1% to 8.2% during the intervention, but there is no significant change in the control group, which indicated that nutrition education can modify parents’ behavior. 

**Table 7:** Whether parents consumed western-style foods (%).

Group	Phase	Yes	No.
Intervention	Initial	13.1 *	86.9
	Metaphase	9.2	90.8
	Final	8.2	91.8
Control	Initial	12.4	87.6
	Metaphase	8.4	91.6
	Final	9.9	90.1

Note: * basd on question: did you consume the western style fast food the last month?

**Table 8:** Consumption frequency of western-style food monthly among parents (%).

Group	Phase	1	2	3	4	5	6	7	8	9 or More
Intervention	Initial	43.6	32.9	11.1	7.6	1.8	0.9	0.4	0.9	0.9
	Metaphase	34.9	27.9	14.0	14.0	3.5	0.0	0.0	1.2	4.7
	Final	42.9	38.3	10.4	6.5	0.0	0.6	0.6	0.0	0.6
Control	Initial	48.6	32.7	10.3	4.7	1.9	0.9	0.0	0.0	0.9
	Metaphase	55.2	24.1	3.4	13.8	0.0	0.0	0.0	0.0	3.4
	Final	53.5	27.9	9.3	7.0	0.0	2.3	0.0	0.0	0.0

## 5. Discussion and Conclusions

The prevalence of obesity in adults and children in developed countries has increased markedly in recent decades, increasing the risk of chronic conditions such as heart disease, diabetes, and cancer, an important contributor to population weight gain has been an increased consumption of foods purchased and prepared out of home such as those from fast food restaurants and supermarkets [14,15]. However, with the economic boom in developing countries like China, the food environment has greatly changed over the last two decades [16]. China consumes fast food at an even faster rate than ever [17]. The success of Kentucky Fried Chicken and McDonald’s in China is the result of a transformation of China’s lifestyles, which is becoming more geared to speed and convenience. Though for now western style fast food has not yet become the dominant dining style in China, it has already put forward a vital challenge to the traditional “dietary culture”, especially for the younger generation. Commercials and other marketing strategies plus the modernized lifestyle have greatly promoted the popularity of western style fast food [18].

In the past years, an abundance of studies have investigated the current western style fast food consumption patterns in China as well as among the Chinese population in other countries. It was reported that compared with the Chinese population in the other countries or areas, mainland Chinese consume less western style fast food. For example, Whitton *et al*. reported that 18% Chinese, 22% Malay, and 22% India regularly consume western style fast food (at least once per week) in Singapore [8]. This could be due to the fact that Singapore is more westernized than the mainland China, which facilitated the access to western style fast food. A survey in England also reported that children living near fast food outlets are more likely to be overweight or obese than those living in other areas [19], indicating that community food environment can greatly affect the dietary behavior of local residents [20]. 

For now, it is hard to evaluate the long-term implications such as diabetes and cardiovascular diseases in Chinese children exposed to the western style fast food, due to the fact that the introduction of western style fast food in mainland China only occurred around a decade ago. western style fast food has already been identified as a leading cause and contributor to many nutrition-related problems in children [21]. However, there is no policy to restrict children from being exposed to western style fast food in China now. Besides, the fact that advertising and food industries oppose regulations to limit advertising to children has greatly interfered with public health policies towards western style fast food, so it becomes important to focus on nutrition education and physical activity strategies [22]. Promoting information about the detrimental effects of western style fast food and establishing healthy dietary habits are critical alternatives for protecting children from fast food. Integrating nutrition education into regular learning activities in the early period of life when dietary habits are established is a feasible and efficient strategy [23,24]. Based on our preliminary observation, implementation of nutrition education can improve both the children’s and their parents’ dietary habits, and we find that children were interested in nutrition education activities [25,26]. 

In present study, we find that one-year nutrition education reduced the western style fast food consumption of parents, but didn’t exert any immediate effect on children. This could be explained by the fact that nutrition education does have beneficial effects, but for children it might take longer to show results, which may due to the fact that children at age of 4–6 years old are more vulnerable to western styles fast food compared with adults. A recently study also found that children’s habits was influenced by their parents’ health awareness. These findings suggest that intervention strategies, even in very young children, should also target parents in order to achieve maximum success [27].

In light of the findings in this study and China’s rapid economic growth, further investigation and increased public health intervention should be conducted since western style fast food is likely to be more and more accessible and affordable in the near future. Our study has several limitations. Information of total energy and food volume were not collected. The long term effects of western style fast food on children is not available since by the time of publication we had just conducted a one-year intervention. However, we employed a large sample size (2102 child-parent pairs) which can sufficiently represent the target population in our study. Additionally, repeated measures of western style fast food behavior in both the intervention and control group make the results more reliable. 

To our knowledge, the current study represents the first community-based intervention study on western style fast food in China. Our study not only provided general information about the consumption of western style fast food in children and their parents in China, but also provides scientific support for efficient policy and guidelines for the restriction of western style fast food [18]. 
